# *LIN28A *gene polymorphisms modify neuroblastoma susceptibility: A four‐centre case‐control study

**DOI:** 10.1111/jcmm.14827

**Published:** 2019-11-20

**Authors:** Rui‐Xi Hua, Zhenjian Zhuo, Lili Ge, Jinhong Zhu, Li Yuan, Chongfen Chen, Jing Liu, Jiwen Cheng, Haixia Zhou, Jiao Zhang, Huimin Xia, Xianwei Zhang, Jing He

**Affiliations:** ^1^ Department of Pediatric Surgery Guangzhou Institute of Pediatrics Guangdong Provincial Key Laboratory of Research in Structural Birth Defect Disease Guangzhou Women and Children's Medical Center Guangzhou Medical University Guangzhou China; ^2^ Department of Oncology The First Affiliated Hospital of Sun Yat‐sen University Guangzhou China; ^3^ Henan Provincial Key Laboratory of Children's Genetics and Metabolic Diseases Children's Hospital Affiliated to Zhengzhou University Henan Children's Hospital Zhengzhou Children's Hospital Zhengzhou China; ^4^ Department of Clinical Laboratory Biobank Harbin Medical University Cancer Hospital Harbin China; ^5^ Department of Pediatric Surgery The Second Affiliated Hospital of Xi'an Jiaotong University Xi'an China; ^6^ Department of Hematology The Second Affiliated Hospital and Yuying Children's Hospital of Wenzhou Medical University Wenzhou China; ^7^ Department of Pediatric Surgery The First Affiliated Hospital of Zhengzhou University Zhengzhou China; ^8^ Department of Pediatric Oncologic Surgery Children's Hospital Affiliated to Zhengzhou University Henan Children's Hospital Zhengzhou Children's Hospital Zhengzhou China

**Keywords:** case‐control study, *LIN28A*, neuroblastoma, polymorphism, risk

## Abstract

Neuroblastoma ranks the most common seen solid tumour in childhood. Overexpression of *LIN28A* gene has been linked to the development of multiple human malignancies, but the relationship between *LIN28A* single nucleotide polymorphisms (SNPs) and neuroblastoma susceptibility is still under debate. Herein, we evaluated the correlation of four potentially functional *LIN28A* SNPs (rs3811464 G>A, rs3811463 T>C, rs34787247 G>A, and rs11247957 G>A) and neuroblastoma susceptibility in 505 neuroblastoma patients and 1070 controls from four independent hospitals in China. The correlation strengths were determined by using odds ratios (ORs) and corresponding 95% confidence intervals (CIs). Among these SNPs, rs34787247 G>A exhibited a significant association with increased susceptibility in neuroblastoma (GA vs GG: adjusted OR = 1.30, 95% CI = 1.03‐1.64; AA vs GG: adjusted OR = 2.51, 95% CI = 1.36‐4.64, AA/GA vs GG: adjusted OR = 1.42, 95% CI = 1.12‐1.80, AA vs GG/GA: adjusted OR = 2.39, 95% CI = 1.29‐4.42). Furthermore, the combined analysis of risk genotypes revealed that subjects carrying three risk genotypes (adjusted OR = 1.64, 95% CI = 1.02‐2.63) are more inclined to develop neuroblastoma than those without risk genotype, and so do carriers of 1‐4 risk genotypes (adjusted OR = 1.26, 95% CI = 1.01‐1.56). Stratification analysis further revealed risk effect of rs3811464 G>A, rs34787247 G>A and 1‐4 risk genotypes in some subgroups. Haplotype analysis of these four SNPs yields two haplotypes significantly correlated with increased neuroblastoma susceptibility. Overall, our finding indicated that *LIN28A* SNPs, especially rs34787247 G>A, may increase neuroblastoma risk.

## 1 INTRODUCTION

Neuroblastoma is a solid tumour that predominantly affects infants and young children.^1^ It mainly develops from neural crest progenitor cells.^2^, ^3^ Neuroblastoma constitutes about 8%‐10% of all paediatric cancers, but disproportionally causes 12%‐15% cancer death in children.^2^, ^4^, ^5^ Neuroblastoma displays considerable clinical heterogeneity, ranging from spontaneous recovery to therapy‐refractory progression.^6^, ^7^ The distinct difference in survival rate among subgroups was another reflection of such heterogeneity.^8^, ^9^ Patients with neuroblastoma can be classified into three risk groups: low risk, intermediate risk and high risk by using some clinical and biological prognostic factors. Patients with a non‐high risk (low and intermediate risk) of neuroblastoma have a long‐term survival rate up to of 90% or above, while those with high risk of neuroblastoma only achieve a survival rate as low as 40%.^10^, ^11^

In recent decades, remarkable advancement has been achieved in comprehending the fundamental aetiology of neuroblastoma.^12^, ^13^ Children's and pregnant women's exposures to many environmental factors were reported to predispose to neuroblastoma, yet the causality could not be finally confirmed.^14^, ^15^ On the other hand, genetic alterations have been shown to be linked to neuroblastoma susceptibility. Mutations in genes *ALK* 16, 17 and *PHOX2B* 18 are frequently observed in hereditary neuroblastoma. Moreover, a number of single nucleotide polymorphisms (SNPs) in association with neuroblastoma predisposition have been identified in genes recently, including *TP53*,^19^ *LIN28B*,^20^ *HACE1*,^20^ *LMO1*,^21^ *BARD1*,^22^ *NEFL* 23 and *CDKN1B*.^24^ Moreover, a fine‐mapping analysis of *BARD1* locus (2q35) also identified two independent genome‐wide neuroblastoma‐associated loci.^25^ However, the present identified genetic variations could not fully account for the carcinogenesis of neuroblastoma. We are still on the discovery journey of unveiling more causative genetic alterations hidden in the bush.

LIN28 is a conserved RNA‐binding protein that plays a significant part in the regulation of cell proliferation, glucose metabolism and pluripotency through interacting with miRNAs.^26^, ^27^ The mammalian genome, *LIN28* gene, encodes two Lin28 paralogs, Lin28A and Lin28B.^28^ *LIN28A* inhibits the maturation process of *let‐7* microRNAs and thereby enhances the translation of *let‐7* target mRNAs.^29^, ^30^ Briefly, cytoplasmic *LIN28A* induces *pre‐let‐7* oligo‐uridylation through a TUTase‐dependent mechanism.^31^ Such poly‐uridylation leads to *pre‐let‐7* instability and eventually reduces the amount of mature *let‐7*.^32^ *let‐7* is a tumour suppressor. Its downregulation promotes tumorigenesis and correlates with poor prognosis.^33^ By binding to a variety of mRNA targets, *LIN28A* also has additional functions except for suppressing *let‐7* maturation.^28^, ^34^

Over‐activation of the *LIN28A* gene has been observed in various human cancers.^35^, ^36^ The mechanism of the *LIN28A*‐mediated tumorigenesis has been extensively investigated. However, the implications of *LIN28A* gene SNPs in neuroblastoma risk remain undiscovered. To determine the relationship between *LIN28A* gene SNPs and neuroblastoma susceptibility, we performed this multi‐centre epidemiological study.

## 2 MATERIALS AND METHODS

### 2.1 Study subjects

The current case‐control study included 505 cases and 1070 healthy non‐cancer controls, as noted previously (Table S1).^37^ Cases were newly diagnosed and histologically confirmed with neuroblastoma. Frequency‐matched controls on age and sex were recruited from the same residing area as cases. Without no exception, every participant provided his/her necessary written informed consent. Demographic information was gathered up by trained interviewers. The complete criterion for selecting participants was addressed in our previous work.^38^ This study has gained its approval from the institutional review boards constituted by all participating hospitals including Guangzhou Women and Children's Medical Center, the First Affiliated Hospital of Zhengzhou University, the Second Affiliated Hospital of Xi'an Jiaotong University, and The Second Affiliated Hospital and Yuying Children's Hospital of Wenzhou Medical University.

### 2.2 Polymorphism selection and genotyping

We retrieved four SNPs with potential function in the *LIN28A* gene from the dbSNP database and SNPinfo software.^39^ Selection criteria were briefly described below: (a) the minor allele frequency reported in HapMap was >5% for Chinese Han subjects; (b) putative functional potentials SNPs located in the 5′‐flanking region, exon, 5′‐untranslated region (UTR) and 3′ UTR, which might affect transcription activity or binding capacity of the microRNA binding site; and (c) SNPs in low linkage disequilibrium (LD) with each other (*R* ^2^ < 0.8). There was no significant LD (*R* ^2^ < 0.8) among these four SNPs of *LIN28A* (*R* ^2^ = 0.183 between rs3811464 and rs3811463, *R* ^2^ = 0.009 between rs3811464 and rs34787247, *R* ^2^ = 0.054 between rs3811464 and rs11247957; *R* ^2^ = 0.03 between rs3811463 and rs34787247, *R* ^2^ = 0.052 between rs3811463 and rs11247957; *R* ^2^ = 0.002 between rs34787247 and rs11247957) (Figure S1). The locations of these SNPs in the *LIN28A* are as below: rs3811464 G>A in the upstream, rs3811463 T>C, rs34787247 G>A, and rs11247957 G>A are all in the 3′ UTR. More detailed selection standards were exhibited at our previous work.^38^ DNA was isolated from the blood sample using a TIANamp Blood DNA Kit (TianGen Biotech Co. Ltd.). Then, the DNA was further performed to genotype using the TaqMan methodology instructed by the manufacturers.^40^, ^41^, ^42^ Negative controls (water samples) were used to ensure genotyping preciseness. A repeated genotyping of 10% randomly selected sample was also conducted in all plates with concordance rates of 100%.

### 2.3 Statistical analysis

The *χ* ^2^ test was applied to test the difference in the distributions of subject characteristics between the cases and controls. A goodness‐of‐fit chi‐squared test was adopted to find whether there exists Hardy‐Weinberg equilibrium (HWE) among controls. Logistic regression analysis was applied to detect any association with neuroblastoma risk, with the crude and adjusted odds ratios (ORs) and 95% confidence intervals (CIs). The adjusted ORs were adjusted for age and gender. We determined the risk genotypes for each SNP based on its association with neuroblastoma risk. If a genotype of a SNP was shown to increase neuroblastoma risk (OR > 1), the genotype was regarded as a risk genotype. Carriers of 3 risk genotypes represented those carrying three risk genotypes of the four SNPs, while 1‐4 risk genotypes represented those carrying 1‐4 risk genotypes.^43^ The stratification analyses were also performed to identify the associations by age, gender, sites of origins and clinical stages. Moreover, a combination of rs3811464 G>A, rs3811463 T>C, rs34787247 G>A, rs11247957 G>A was regarded as a haplotype. Unphased genotype data were used to identify haplotype frequencies and individual haplotypes. Logistic regression analyses also help to obtain haplotype frequencies and distinct haplotypes, with the adjustment for gender and age.^44^, ^45^ The haplotype of the highest rate was used as the reference group to calculate ORs for haplotype associated with neuroblastoma risk. We set *P* < .05 as a significant borderline for all tests. We used SAS 9.1 (SAS Institute, Cary, NC) to compute all statistics.

## 3 RESULTS

### 3.1 The relationship between *LIN28A* SNPs and neuroblastoma susceptibility

The association of all variant genotypes of the four *LIN28A* SNPs (rs3811464 G>A, rs3811463 T>C, rs34787247 G>A, rs11247957 G>A) with neuroblastoma risk is shown in Table 1 for combined subjects and in Table S2 for divided subjects. All these SNPs in controls were in accordance with HWE (all with an HWE *P* > .05). In the single‐locus analysis, only one variant, rs34787247 G>A, in the *LIN28A* gene could significantly influence neuroblastoma susceptibility. Carriers of rs34787247A allele showed increased susceptibility to neuroblastoma (GA vs GG: adjusted OR = 1.30, 95% CI = 1.03‐1.64, *P* = .027; AA vs GG: adjusted OR = 2.51, 95% CI = 1.36‐4.64, *P* = .003; AA/GA vs GG: adjusted OR = 1.42, 95% CI = 1.12‐1.80, *P* = .004; AA vs GG/GA: adjusted OR = 2.39, 95% CI = 1.29‐4.42, *P* = .006). The rs3811464 GA/AA, rs3811463 TC/CC, rs34787247 GA/AA and rs11247957 GA/AA are treated as risk genotypes. Then, we analysed the combined effect of risk genotypes and observed that participants with 3 or 1‐4 risk genotypes experienced a 1.64‐fold (adjusted OR = 1.64, 95% CI = 1.02‐2.63, *P* = .04) and 1.26‐fold (adjusted OR = 1.26, 95% CI = 1.01‐1.56, *P* = .04) increase in the risk of developing neuroblastoma, respectively.

**Table 1:** Association between *LIN28A* gene polymorphisms and neuroblastoma susceptibility

Genotype	Cases (N = 505)	Controls (N = 1070)	*P* a	Crude OR (95% CI)	*P*	Adjusted OR (95% CI)^b^	*P* b
	No. (%)	No. (%)					
rs3811464 G>A (HWE = 0.063)							
GG	359 (71.09)	790 (73.83)		1.00		1.00	
GA	123 (24.36)	250 (23.36)		1.10 (0.87‐1.39)	.440	1.09 (0.87‐1.38)	.452
AA	23 (4.55)	30 (2.80)		1.71 (0.99‐2.96)	.057	1.70 (0.98‐2.96)	.058
Additive			.115	1.17 (0.96‐1.43)	.116	1.17 (0.96‐1.43)	.114
Dominant	146 (28.91)	280 (26.17)	.253	1.15 (0.91‐1.45)	.253	1.15 (0.91‐1.45)	.250
Recessive	482 (95.45)	1040 (97.20)	.072	1.65 (0.95‐2.88)	.075	1.66 (0.95‐2.88)	.075
rs3811463 T>C (HWE = 0.530)							
TT	364 (72.08)	785 (73.36)		1.00		1.00	
TC	127 (25.15)	260 (24.30)		1.08 (0.86‐1.36)	.528	1.07 (0.85‐1.35)	.558
CC	14 (2.77)	25 (2.34)		1.24 (0.64‐2.39)	.531	1.25 (0.64‐2.41)	.514
Additive			.530	1.07 (0.87‐1.31)	.530	1.07 (0.87‐1.32)	.525
Dominant	141 (27.92)	285 (26.64)	.592	1.07 (0.84‐1.35)	.592	1.07 (0.84‐1.35)	.591
Recessive	491 (97.23)	1045 (97.66)	.603	1.19 (0.61‐2.31)	.604	1.20 (0.62‐2.33)	.591
rs34787247 G>A (HWE = 0.390)							
GG	353 (69.90)	821 (76.73)		1.00		1.00	
GA	130 (25.74)	229 (21.40)		**1.30 (1.03‐1.65)**	**.026**	**1.30 (1.03‐1.64)**	**.027**
AA	22 (4.36)	20 (1.87)		**2.52 (1.37‐4.66)**	**.003**	**2.51 (1.36‐4.64)**	**.003**
Additive			.0006	**1.42 (1.16‐1.74)**	**.0007**	**1.42 (1.16‐1.74)**	**.0007**
Dominant	152 (30.10)	249 (23.27)	.004	**1.42 (1.12‐1.80)**	**.004**	**1.42 (1.12‐1.80)**	**.004**
Recessive	483 (95.64)	1050 (98.13)	.004	**2.39 (1.29‐4.42)**	**.006**	**2.39 (1.29‐4.42)**	**.006**
rs11247957 G>A (HWE = 0.554)							
GG	481 (95.25)	1032 (96.45)		1.00		1.00	
GA	24 (4.75)	38 (3.55)		1.38 (0.83‐2.32)	.219	1.39 (0.83‐2.33)	.218
AA	0 (0.00)	0 (0.00)		/	/	/	/
Additive			.253	1.36 (0.80‐2.29)	.254	1.36 (0.81‐2.30)	.249
Dominant	24 (4.75)	38 (3.55)	.253	1.36 (0.80‐2.29)	.254	1.36 (0.81‐2.30)	.249
Combined effect of risk genotypes^c^							
0	192 (38.02)	465 (43.46)		1.00		1.00	
1	198 (39.21)	404 (37.76)		1.14 (0.93‐1.39)	.204	1.14 (0.93‐1.39)	.213
2	82 (16.24)	156 (14.58)		1.22 (0.92‐1.63)	.173	1.21 (0.91‐1.62)	.188
3	31 (6.14)	44 (4.11)		**1.64 (1.02‐2.62)**	**.041**	**1.64 (1.02‐2.63)**	**.040**
4	2 (0.40)	1 (0.09)		4.64 (0.42‐51.33)	.210	4.54 (0.42‐50.24)	.217
Trend			.010	**1.17 (1.04‐1.32)**	**.010**	**1.17 (1.04‐1.32)**	**.010**
0	192 (38.02)	465 (43.46)		1.00		1.00	
1‐4	313 (61.98)	605 (56.54)	.041	**1.25 (1.01‐1.56)**	**.041**	**1.26 (1.01‐1.56)**	**.040**

Abbreviations: CI, confidence interval; OR, odds ratio.

Significance of bold values are the P values less than 0.05 or the 95% CIs excluded 1.

a *χ* ^2^ test for genotype distributions between neuroblastoma patients and controls.

b Adjusted for age and gender.

c Risk genotypes were rs3811464 GA/AA, rs3811463 TC/CC, rs34787247 GA/AA and rs11247957 GA/AA.

### 3.2 Stratification analysis

Table 2 displays the contents of the association between *LIN28A* gene polymorphisms and susceptibility to neuroblastoma in certain groups separated by age, gender, sites of origins and clinical stages. We detected the rs3811464 AA genotypes carriers were more likely to have increased neuroblastoma risk in the subgroup of tumours in retroperitoneal (adjusted OR = 2.23, 95% CI = 1.04‐4.81, *P* = .041). As for rs34787247 polymorphism, compared to its GG genotype, stronger risk effect of GA/AA genotypes was found among children ≤18 month (adjusted OR = 1.51, 95% CI = 1.04‐2.19, *P* = .031), >18 month (adjusted OR = 1.38, 95% CI = 1.01‐2.87, *P* = .042), males (adjusted OR = 1.47, 95% CI = 1.08‐2.01, *P* = .015) and patients at clinical stages of III + IV (adjusted OR = 1.43, 95% CI = 1.05‐1.97, *P* = .025). Besides, the combined analysis stated that the 1‐4 risk genotypes had an enhanced neuroblastoma risk in the patients with tumour in retroperitoneal (adjusted OR = 1.44, 95% CI = 1.003‐2.07, *P* = .048) and subgroup at early clinical stages I + II + 4S (adjusted OR = 1.44, 95% CI = 1.08‐1.91, *P* = .014).

**Table 2:** Stratification analysis of risk genotypes with neuroblastoma susceptibility

Variables	rs3811464 (cases/controls)		AOR (95% CI)^a^	*P* a	rs34787247 (cases/controls)		AOR (95% CI)^a^	*P* a	Combined risk genotypes (case/controls)		AOR (95% CI)^a^	*P* a
	GG/GA	AA			GG	GA/AA			0	1‐4		
Age, month												
≤18	181/413	8/12	1.50 (0.60‐3.75)	.381	126/319	63/106	**1.51 (1.04‐2.19)**	**.031**	67/186	122/239	1.42 (0.99‐2.02)	.055
>18	301/627	15/18	1.74 (0.87‐3.51)	.120	227/502	89/143	**1.38 (1.01‐1.87)**	**.042**	125/279	191/366	1.17 (0.89‐1.54)	.271
Gender												
Females	203/439	10/9	2.41 (0.97‐6.04)	.060	149/340	64/108	1.35 (0.94‐1.95)	.105	81/200	132/248	1.31 (0.94‐1.83)	.109
Males	279/601	13/21	1.34 (0.66‐2.72)	.415	204/481	88/141	**1.47 (1.08‐2.01)**	**.015**	111/265	181/357	1.21 (0.91‐1.61)	.184
Sites of origin												
Adrenal gland	167/1040	6/30	1.28 (0.52‐3.13)	.593	124/821	49/249	1.31 (0.91‐1.88)	.147	69/465	104/605	1.17 (0.84‐1.62)	.359
Retroperitoneal	138/1040	9/30	**2.23 (1.04‐4.81)**	**.041**	105/821	42/249	1.33 (0.90‐1.95)	.152	51/465	96/605	**1.44 (1.003‐2.07)**	**.048**
Mediastinum	129/1040	6/30	1.67 (0.68‐4.11)	.262	94/821	41/249	1.43 (0.96‐2.12)	.078	55/465	80/605	1.13 (0.78‐1.62)	.528
Others	40/1040	2/30	1.67 (0.38‐7.27)	.494	27/821	15/249	1.84 (0.96‐3.51)	.066	15/465	27/605	1.38 (0.73‐2.63)	.326
Clinical stages												
I + II + 4s	237/1040	13/30	1.93 (0.99‐3.75)	.054	177/821	73/249	1.35 (1.00‐1.84)	.054	87/465	163/605	**1.44 (1.08‐1.91)**	**.014**
III + IV	222/1040	10/30	1.59 (0.76‐3.31)	.216	162/821	70/249	**1.43 (1.05‐1.97)**	**.025**	97/465	135/605	1.07 (0.80‐1.43)	.646

Abbreviations: AOR, adjusted odds ratio; CI, confidence interval.

Significance of bold values are the P values less than 0.05 or the 95% CIs excluded 1.

a Adjusted for age and gender, omitting the corresponding stratify factor.

### 3.3 *LIN28A* haplotype analysis

We further determined whether the haplotypes of the four *LIN28A* SNPs were linked to neuroblastoma risk. As shown in Table 3, the haplotype consisting of wild‐type alleles (GTGG) was defined as the reference group. We detected a significant elevated neuroblastoma risk in subjects with haplotypes GTAG (adjusted OR = 1.35, 95% CI = 1.07‐1.72, *P* = .012) and ACAG (adjusted OR = 3.20, 95% CI = 1.41‐7.25, *P* = .005).

**Table 3:** Association between inferred haplotypes of *LIN28A* gene and neuroblastoma susceptibility

Haplotypes^a^	Cases (N = 1010)	Controls (N = 2140)	Crude OR (95% CI)	*P*	Adjusted OR^b^ (95% CI)	*P* b
	No. (%)	No. (%)				
GTGG	642 (63.56)	1467 (68.55)	1.00		1.00	
GTGA	2 (0.20)	0 (0.00)	/	/	/	/
GTAG	129 (12.77)	218 (10.19)	**1.35 (1.07‐1.71)**	**.013**	**1.35 (1.07‐1.72)**	**.012**
GTAA	1 (0.10)	0 (0.00)	/	/	/	/
GCGG	54 (5.35)	123 (5.75)	1.00 (0.72‐1.40)	.985	1.01 (0.72‐1.40)	.977
GCGA	2 (0.20)	3 (0.14)	1.52 (0.25‐9.14)	.645	1.51 (0.25‐9.04)	.655
GCAG	10 (0.99)	18 (0.84)	1.27 (0.58‐2.77)	.548	1.27 (0.58‐2.76)	.551
GCAA	1 (0.10)	1 (0.05)	2.29 (0.14‐36.59)	.559	2.29 (0.14‐36.71)	.558
ATGG	64 (6.34)	124 (5.79)	1.18 (0.86‐1.62)	.306	1.18 (0.86‐1.62)	.304
ATGA	0 (0.00)	0 (0.00)	/	/	/	/
ATAG	17 (1.68)	21 (0.98)	1.85 (0.97‐3.53)	.062	1.85 (0.97‐3.53)	.062
ATAA	0 (0)	0 (0.00)	/	/	/	/
ACGG	56 (5.54)	121 (5.65)	1.06 (0.76‐1.47)	.740	1.06 (0.76‐1.47)	.740
ACGA	16 (1.58)	33 (1.54)	1.11 (0.61‐2.03)	.740	1.11 (0.61‐2.04)	.728
ACAG	14 (1.39)	10 (0.47)	**3.20 (1.41‐7.24)**	**.005**	**3.20 (1.41‐7.25)**	**.005**
ACAA	2 (0.20)	1 (0.05)	4.57 (0.41‐50.49)	.215	4.54 (0.41‐50.19)	.217

Abbreviations: CI, confidence interval; OR, odds ratio.

Significance of bold values are the P values less than 0.05 or the 95% CIs excluded 1.

a The haplotype order was rs3811464, rs3811463, rs34787247 and rs11247957.

b Obtained in logistic regression models with adjustment for age and gender.

## 4 DISCUSSION

At the present, there remain many hidden genetic factors in association with neuroblastoma risk to be discovered to fill up the knowledge gaps. Thus, the identification of more polymorphisms is needed to unearth the full range of neuroblastoma susceptibility variations. Herein, we undertook a four‐centre case‐control study to investigate the role of *LIN28A* polymorphisms on neuroblastoma risk in Chinese children. We are the pioneer in unveiling the association of the rs34787247A allele with an elevated neuroblastoma risk in a Chinese population.

*LIN28A* gene resides on chromosome 1p36.11. Several lines of evidence suggested the roles of *LIN28A* gene polymorphisms in cancer risk. Zhang et al^46^ found *LIN28B* rs221636 could decrease the risk of oral cavity cancer in a study of 384 cases and 731 healthy controls, including six SNPs in *let‐7*/*LIN28* gene. Nevertheless, they failed to detect the association of *LIN28A* rs4659441 and rs3811463 with oral cavity cancer risk. Permuth‐Wey et al^47^ observed the predisposing role of rs12728900 and rs11247946 in *LIN28A* on epithelial ovarian cancer in European ancestry. Sung et al^48^ carried out two genome‐wide association studies in East Asia, 5066 breast cancer cases and 4337 controls recruited from Chinese and Koreans. They reported that the 237 SNPs located in microRNA biogenesis pertinent pathway genes had no significant association with breast cancer risk, including SNPs of *LIN28A* (rs11247954, rs12728900, rs3811463, rs4274112, rs4659441, rs6598964, and rs6683792). Chen et al^49^ determined the effect of SNPs of *LIN28* gene on the breast cancer risk. In analysing five SNPs (rs12122703 A>G, rs3811464 G>A, rs11247955 G>A, rs3811463 T>C, rs6697410 T>G) in *LIN28*, they successfully identified rs3811463 and rs6697410 to be linked to breast cancer risk using a hospital‐based case‐control study in 1004 cases and 1296 controls. They further conducted a community‐based validation study using 511 cases and 645 controls. They validated that the rs3811463‐C allele predisposed to an increased risk of breast cancer. Further functional experiments suggested that the rs3811463C allele located near the *let‐7* binding site of the *LIN28* gene. This variant attenuated *let‐7*‐induced degradation of *LIN28* mRNA, leading to enhanced levels of LIN28 protein, which could, in turn, decrease mature *let‐7* level, finally alter breast cancer risk. The quite association results of *LIN28A* SNPs reported by studies implied that the effects of *LIN28A* on cancer risk would be modified by many factors like ethnicities, sample sizes and cancer types. Therefore, discovering the function of *LIN28A* SNPs on a particular type of cancer and specific ethnicity is of great necessity.

Given *LIN28A*'s vital role in malignancies, we undertook this first epidemiological study to outline the correlation of *LIN28A* polymorphisms and neuroblastoma risk in a Chinese population. Despite the abundance of reports on *LIN28A* gene variation and cancers, investigations of contribution of *LIN28A* SNPs to neuroblastoma cancer risks were scarce. Our results thoroughly showed rs34787247A allele could contribute to an increased neuroblastoma risk. Moreover, we also observed an enhanced risk of neuroblastoma in subjects carrying 3 or 1‐4 risk genotypes. However, no significant relationship of the rest three variants rs3811464, rs3811463 and rs11247957 was detected.

We carried out a pioneering study on the association between *LIN28A* gene SNPs and susceptibility to neuroblastoma. Limitations also existed. Firstly, the sample size is not large enough to generate reliable statistics. Some of the results might be merely fortuitous events, particularly the stratification analysis. Secondly, we examined four SNPs in this research. More potential neuroblastoma risk‐associated SNPs in the *LIN28A* gene await to be explored. Thirdly, although the participants were enrolled from four different cities, findings from the restricted Chinese population could not be extrapolated to other ethnicities directly. Lastly, environmental factors were not considered in this study.

In all, we presented a multi‐centre case‐control study in Chinese children. For the first time, our findings unveiled a contributing role of *LIN28A* gene SNPs in neuroblastoma risk. In the future, an integrative analysis, covering more profound and specific factors, with environmental factors, genetic‐environmental interaction, should be carried out to unearth the aetiology of neuroblastoma.

## CONFLICT OF INTEREST

The authors declare no competing financial interests.

## AUTHOR'S CONTRIBUTIONS

All authors contributed significantly to this work. RX Hua, L. Yuan, C. Chen, J. Liu, J. Cheng, H. Zhou, J. Zhang, and J. He performed the research study and collected the data; J. He analysed the data; H. Xia, X. Zhang and J. He designed the research study; RX Hua, Z. Zhuo and L. Ge wrote the paper; J Zhu and J. He prepared all the Tables. All authors reviewed the manuscript. In addition, all authors have read and approved the manuscript.

## Data Availability

All the data were available upon request.
