# Supplementary material for: LIN28A gene polymorphisms modify neuroblastoma susceptibility: A four‐centre case‐control study
Source: J Cell Mol Med. 2019 Nov 20;24(1):1059–66. doi: 10.1111/jcmm.14827 (PMC6933387; doi:10.1111/jcmm.14827)
Supplement: Supplementary file 1 [file JCMM-24-1059-s001.doc]

| **Supplemental Table S1**. Frequency distribution of selected characteristics in cases and controls | | | | | | | |  | |
| --- | --- | --- | --- | --- | --- | --- | --- | --- | --- |
| Variables | Cases (N=505) | | Controls (N=1070) | | | *P* ^a^ | | |  |
|  | No. | % | | No. | % | |  | | |
| Age range, month | 0.00-132.00 | | 0.03-156.00 | | | 0.384 | | |  |
| Mean ± SD | 33.08±27.38 | | 32.27±26.89 | | |  | | |  |
| ≤18 | 189 | 37.43 | | 425 | 39.72 | |  | | |
| >18 | 316 | 62.57 | | 645 | 60.28 | |  | | |
| Gender |  |  | |  |  | | 0.908 | | |
| Female | 213 | 42.18 | | 448 | 41.87 | |  | | |
| Male | 292 | 57.82 | | 622 | 58.13 | |  | | |
| INSS stages |  |  | |  |  | |  | | |
| I | 148 | 29.31 | | / | / | |  | | |
| II | 102 | 20.20 | | / | / | |  | | |
| III | 78 | 15.45 | | / | / | |  | | |
| IV | 154 | 30.50 | | / | / | |  | | |
| 4s | 14 | 2.77 | | / | / | |  | | |
| NA | 9 | 1.78 | | / | / | |  | | |
| Sites of origin |  |  | |  |  | |  | | |
| Adrenal gland | 173 | 34.26 | | / | / | |  | | |
| Retroperitoneal region | 147 | 29.11 | | / | / | |  | | |
| Mediastinum | 135 | 26.73 | | / | / | |  | | |
| Other region | 42 | 8.32 | | / | / | |  | | |
| NA | 8 | 1.58 | | / | / | |  | | |
| SD, standard deviation; NA, not available.  ^a^ Two-sided χ^2^ test for distributions between neuroblastoma cases and cancer-free controls. | | | | | | | |  | |

| **Supplemental Table S2**. Logistic regression analysis for the association between *LIN28A* gene polymorphisms and neuroblastoma risk for divided subjects | | | | | | | | | | | | | | | | |
| --- | --- | --- | --- | --- | --- | --- | --- | --- | --- | --- | --- | --- | --- | --- | --- | --- |
| Genotype | Guangdong province | | | | Henan province | | | | Wenzhou area | | | | Shaanxi province | | | |
|  | Cases  (N=275) | Controls  (N=531) | AOR (95% CI) ^a^ | *P* ^a^ | Cases  (N=118) | Controls  (N=281) | AOR (95% CI) ^a^ | *P* ^a^ | Cases  (N=36) | Controls  (N=72) | AOR (95% CI) ^a^ | *P* ^a^ | Cases  (N=76) | Controls  (N=186) | AOR (95% CI) ^a^ | *P* ^a^ |
|  | No. (%) | No. (%) |  |  | No. (%) | No. (%) |  |  | No. (%) | No. (%) |  |  | No. (%) | No. (%) |  |  |
| rs3811464 G>A | | | | | | | | | | | | | | | | |
| GG | 206 (74.91) | 402 (75.71) | 1.00 |  | 83 (70.34) | 217 (77.22) | 1.00 |  | 23 (63.89) | 43 (59.72) | 1.00 |  | 47 (61.84) | 128 (68.82) | 1.00 |  |
| GA | 56 (20.36) | 116 (21.85) | 0.96 (0.67-1.37) | 0.802 | 30 (25.42) | 57 (20.28) | 1.39 (0.83-2.33) | 0.205 | 11 (30.56) | 26 (36.11) | 0.79 (0.33-1.89) | 0.592 | 26 (34.21) | 51 (27.42) | 1.39 (0.78-2.48) | 0.266 |
| AA | 13 (4.73) | 13 (2.45) | 1.98 (0.90-4.35) | 0.090 | 5 (4.24) | 7 (2.49) | 2.05 (0.63-6.69) | 0.236 | 2 (5.56) | 3 (4.17) | 1.34 (0.20-8.77) | 0.762 | 3 (3.95) | 7 (3.76) | 1.13 (0.28-4.61) | 0.860 |
| Additive |  |  | 1.13 (0.86-1.50) | 0.381 |  |  | 1.41 (0.94-2.12) | 0.100 |  |  | 0.93 (0.46-1.88) | 0.842 |  |  | 1.25 (0.78-1.99) | 0.357 |
| Dominant | 69 (25.09) | 129 (24.29) | 1.06 (0.75-1.48) | 0.745 | 35 (29.66) | 64 (22.78) | 1.46 (0.90-2.38) | 0.128 | 13 (36.11) | 29 (40.28) | 0.84 (0.37-1.93) | 0.684 | 29 (38.16) | 58 (31.18) | 1.36 (0.78-2.37) | 0.282 |
| Recessive | 262 (95.27) | 518 (97.55) | 2.00 (0.91-4.37) | 0.084 | 113 (95.76) | 274 (97.51) | 1.89 (0.58-6.12) | 0.291 | 34 (94.44) | 69 (95.83) | 1.46 (0.23-9.31) | 0.692 | 73 (96.05) | 179 (96.24) | 1.02 (0.26-4.10) | 0.975 |
| rs3811463 T>C | | | | | | | | | | | | | | | | |
| TT | 222 (80.73) | 396 (74.58) | 1.00 |  | 78 (66.10) | 213 (75.80) | 1.00 |  | 22 (61.11) | 48 (66.67) | 1.00 |  | 42 (55.26) | 128 (68.82) | 1.00 |  |
| TC | 49 (17.82) | 121 (22.79) | 0.72 (0.50-1.04) | 0.080 | 37 (31.36) | 65 (23.13) | 1.60 (0.98-2.59) | 0.058 | 12 (33.33) | 21 (29.17) | 1.22 (0.51-2.94) | 0.650 | 29 (38.16) | 53 (28.49) | 1.66 (0.94-2.94) | 0.083 |
| CC | 4 (1.45) | 14 (2.64) | 0.51 (0.17-1.58) | 0.245 | 3 (2.54) | 3 (1.07) | 2.87 (0.56-14.59) | 0.204 | 2 (5.56) | 3 (4.17) | 1.31 (0.20-8.54) | 0.778 | 5 (6.58) | 5 (2.69) | 3.04 (0.82-11.26) | 0.095 |
| Additive |  |  | **0.72 (0.52-0.99)** | **0.041** |  |  | **1.62 (1.05-2.49)** | **0.028** |  |  | 1.19 (0.60-2.36) | 0.625 |  |  | **1.70 (1.07-2.69)** | **0.026** |
| Dominant | 53 (19.27) | 135 (25.42) | **0.70 (0.49-1.00)** | **0.048** | 40 (33.90) | 68 (24.20) | **1.65 (1.03-2.65)** | **0.037** | 14 (38.89) | 24 (33.33) | 1.24 (0.54-2.85) | 0.620 | 34 (44.74) | 58 (31.18) | **1.78 (1.03-3.08)** | **0.040** |
| Recessive | 271 (98.55) | 517 (97.36) | 0.55 (0.18-1.69) | 0.295 | 115 (97.46) | 278 (98.93) | 2.52 (0.50-12.70) | 0.265 | 34 (94.44) | 69 (95.83) | 1.22 (0.19-7.78) | 0.831 | 71 (93.42) | 181 (97.31) | 2.57 (0.71-9.34) | 0.153 |
| rs34787247 G>A | | | | | | | | | | | | | | | | |
| GG | 177 (64.36) | 379 (71.37) | 1.00 |  | 94 (79.66) | 232 (82.56) | 1.00 |  | 24 (66.67) | 52 (72.22) | 1.00 |  | 58 (76.32) | 158 (84.95) | 1.00 |  |
| GA | 84 (30.55) | 136 (25.61) | 1.32 (0.96-1.83) | 0.091 | 19 (16.10) | 49 (17.44) | 0.94 (0.53-1.69) | 0.837 | 11 (30.56) | 20 (27.78) | 1.13 (0.46-2.78) | 0.794 | 16 (21.05) | 24 (12.90) | 1.83 (0.91-3.70) | 0.091 |
| AA | 14 (5.09) | 16 (3.01) | 1.84 (0.88-3.87) | 0.105 | 5 (4.24) | 0 (0.00) | / | / | 1 (2.78) | 0 (0.00) | / | / | 2 (2.63) | 4 (2.15) | 1.33 (0.24-7.49) | 0.749 |
| Additive |  |  | **1.34 (1.03-1.74)** | **0.028** |  |  | 1.44 (0.89-2.33) | 0.140 |  |  | 1.33 (0.57-3.10) | 0.504 |  |  | 1.52 (0.87-2.64) | 0.144 |
| Dominant | 98 (35.64) | 152 (28.63) | **1.38 (1.01-1.88)** | **0.042** | 24 (20.34) | 49 (17.44) | 1.19 (0.69-2.06) | 0.526 | 12 (33.33) | 20 (27.78) | 1.21 (0.50-2.94) | 0.675 | 18 (23.68) | 28 (15.05) | 1.76 (0.91-3.42) | 0.096 |
| Recessive | 261 (94.91) | 515 (96.99) | 1.70 (0.82-3.54) | 0.157 | 113 (95.76) | 281 (100.00) | / | / | 35 (97.22) | 72 (100.00) | / | / | 74 (97.37) | 182 (97.85) | 1.20 (0.21-6.76) | 0.833 |
| rs11247957 G>A | | | | | | | | | | | | | | | | |
| GG | 264 (96.00) | 513 (96.61) | 1.00 |  | 115 (97.46) | 277 (98.58) | 1.00 |  | 31 (86.11) | 66 (91.67) | 1.00 |  | 71 (93.42) | 176 (94.62) | 1.00 |  |
| GA | 11 (4.00) | 18 (3.39) | 1.16 (0.54-2.51) | 0.698 | 3 (2.54) | 4 (1.42) | 1.85 (0.41-8.45) | 0.427 | 5 (13.89) | 6 (8.33) | 2.01 (0.55-7.38) | 0.293 | 5 (6.58) | 10 (5.38) | 1.21 (0.39-3.72) | 0.742 |
| AA | 0 (0.00) | 0 (0.0) | / | / | 0 (0.00) | 0 (0.00) | / | / | 0 (0.00) | 0 (0.00) | / | / | 0 (0.00) | 0 (0.00) | / | / |
| Additive |  |  | 1.16 (0.54-2.51) | 0.698 |  |  | 1.85 (0.41-8.45) | 0.427 |  |  | 2.01 (0.55-7.38) | 0.293 |  |  | 1.21 (0.39-3.72) | 0.742 |
| Dominant | 11 (4.00) | 18 (3.39) | 1.16 (0.54-2.51) | 0.698 | 3 (2.54) | 4 (1.42) | 1.85 (0.41-8.45) | 0.427 | 5 (13.89) | 6 (8.33) | 2.01 (0.55-7.38) | 0.293 | 5 (6.58) | 10 (5.38) | 1.21 (0.39-3.72) | 0.742 |
| Combined effect of risk genotypes ^b^ | | | | | | | | | | | | | | | | |
| 0 | 110 (40.00) | 220 (41.43) | 1.00 |  | 53 (44.92) | 143 (50.89) | 1.00 |  | 10 (27.78) | 22 (30.56) | 1.00 |  | 19 (25.00) | 80 (43.01) | 1.00 |  |
| 1 | 115 (41.82) | 211 (39.74) | 1.09 (0.79-1.51) | 0.594 | 35 (29.66) | 95 (33.81) | 1.00 (0.60-1.64) | 0.989 | 12 (33.33) | 29 (40.28) | 0.89 (0.32-2.51) | 0.832 | 36 (47.37) | 69 (37.10) | **2.20 (1.15-4.19)** | **0.017** |
| 2 | 35 (12.73) | 77 (14.50) | 0.92 (0.58-1.45) | 0.706 | 23 (19.49) | 39 (13.88) | 1.64 (0.89-3.01) | 0.114 | 10 (27.78) | 14 (19.44) | 1.45 (0.46-4.55) | 0.521 | 14 (18.42) | 26 (13.98) | 2.25 (0.99-5.12) | 0.053 |
| 3 | 14 (5.09) | 23 (4.33) | 1.22 (0.60-2.46) | 0.581 | 7 (5.93) | 4 (1.42) | **5.01 (1.40-17.92)** | **0.013** | 4 (11.11) | 6 (8.33) | 1.50 (0.34-6.57) | 0.593 | 6 (7.89) | 11 (5.91) | 2.27 (0.74-6.93) | 0.151 |
| 4 | 1 (0.36) | 0 (0.00) | / | / | 0 (0.00) | 0 (0.00) | / | / | 0 (0.00) | 1 (1.39) | / | / | 1 (1.32) | 0 (0.00) | / | / |
| Trend |  |  | 1.03 (0.87-1.23) | 0.710 |  |  | **1.36 (1.05-1.76)** | **0.019** |  |  | 1.12 (0.75-1.70) | 0.578 |  |  | **1.43 (1.07-1.92)** | **0.016** |
| 0 | 110 (40.00) | 220 (41.43) | 1.00 |  | 53 (44.92) | 143 (50.89) | 1.00 |  | 10 (27.78) | 22 (30.56) | 1.00 |  | 19 (25.00) | 80 (43.01) | 1.00 |  |
| 1-4 | 165 (60.00) | 311 (58.57) | 1.06 (0.79-1.43) | 0.682 | 65 (55.08) | 138 (49.11) | 1.28 (0.83-1.98) | 0.259 | 26 (72.22) | 50 (69.44) | 1.10 (0.44-2.74) | 0.835 | 57 (75.00) | 106 (56.99) | **2.27 (1.25-4.11)** | **0.007** |
| OR, odds ratio; CI, confidence interval.  ^a^ Adjusted for age and gender.  ^b^ Risk genotypes were rs3811464 GA/AA, rs3811463 TC/CC, rs34787247 GA/AA and rs11247957 GA/AA, derived from combined subjects. | | | | | | | | | | | | | | | | |


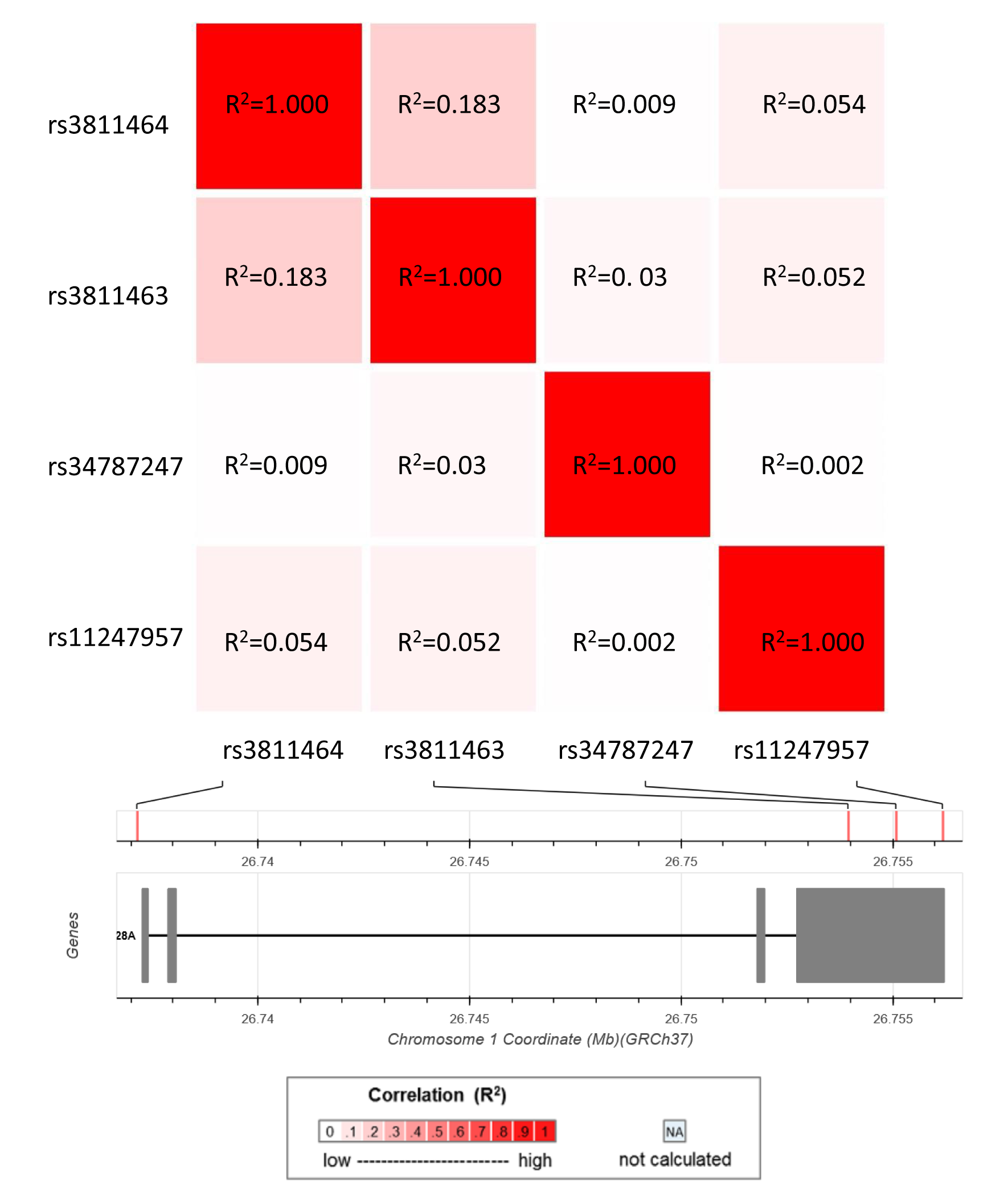


**Supplementary Figure S1**. Linkage disequilibrium (LD) analysis for the four selected SNPs in the Chinese Han population consisting of CHB (Han Chinese in Beijing, China) and CHS (Southern Han Chinese) participants. LD as R^2^ for SNP pairs is shown inside the squares.
